# Joint venture surgery of a giant inguinoscrotal hernia in a patient suffering from trisomy 21 and Eisenmenger's syndrome

**DOI:** 10.1016/j.eucr.2021.101757

**Published:** 2021-06-20

**Authors:** Anne Sofie Virring Brandt, Julie Tastesen, Mille Sværdborg, Hans Jørgen Kirkeby

**Affiliations:** a Department of Urology, Aarhus University Hospital, Aarhus, Denmark; b Department of Plastic and Breast Surgery, Aarhus University Hospital, Aarhus, Denmark

**Keywords:** Inguinoscrotal hernia, Eisenmenger's syndrome, Trisomy 21, ITU, Intensive care unit, GISH, giant inguinoscrotal hernias

## Abstract

A 44-year-old man with Trisomy 21 and Eisenmenger's syndrome underwent surgery due to a life-threatening scrotal hernia, containing the bladder, bilateral hydroceles and part of the sigmoid colon. Joint venture plastic and urologic surgery was performed with reposition of the bladder and sigmoid colon into the abdominal cavity, bilateral inguinal hernial mesh repair, left sided orchiectomy, excision of bilateral hydroceles and excision of a major part of the scrotum and recreation of the original anatomy of the penis and scrotum. This Case presents a successful outcome achieved as the patient after one-step-surgery was left to normal condition.

## Introduction

Giant inguinoscrotal hernias (GISH) are defined as hernias extending below the midpoint of the inner thigh in standing position. GISH are uncommon in modern surgical practice but can be seen as a result of neglect, fear of surgical complications or massive comorbidity.^1^ Complications of GISH include urinary retention, infection and incarceration. We will describe the successful surgical management of a GISH in a patient with Trisomy 21 and Eisenmenger's syndrome.

## Case presentation

This 44-year-old patient, BMI 33, with GISH and Trisomy 21 and Eisenmenger's syndrome was first seen at the Department of Urology at Aarhus University Hospital in November 2019. The patient had two big inguinal hernias which had expanded the scrotum and buried the penis, causing incontinence and incomplete bladder emptying. Due to these difficulties a permanent indwelling transurethral was inserted, but because of the very difficult urethral access the regular change of catheter had to be performed under use of a flexible cystoscope every third month, causing lots of discomfort to him.

**Fig. 1:**
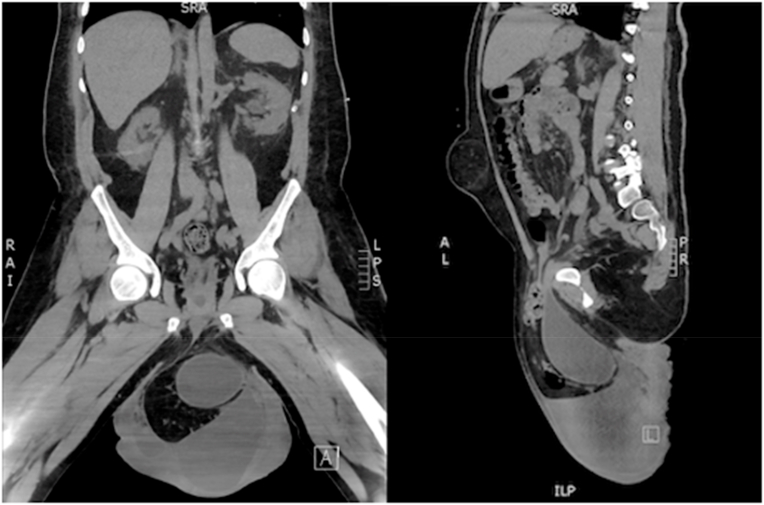
CT-scan before surgery, showing majority of the bladder, a part of the sigmoid colon and some of the omentum situated in the left inguinal hernia. Besides the above, the patient had two big hydroceles, and bilaterally there were hydronephrosis and hydroureter, which were immediately relieved with two nephrostomy catheters. The scan also shows an umbilical hernia which did not represent any threat to the patient and was left untreated.

**Fig. 2:**
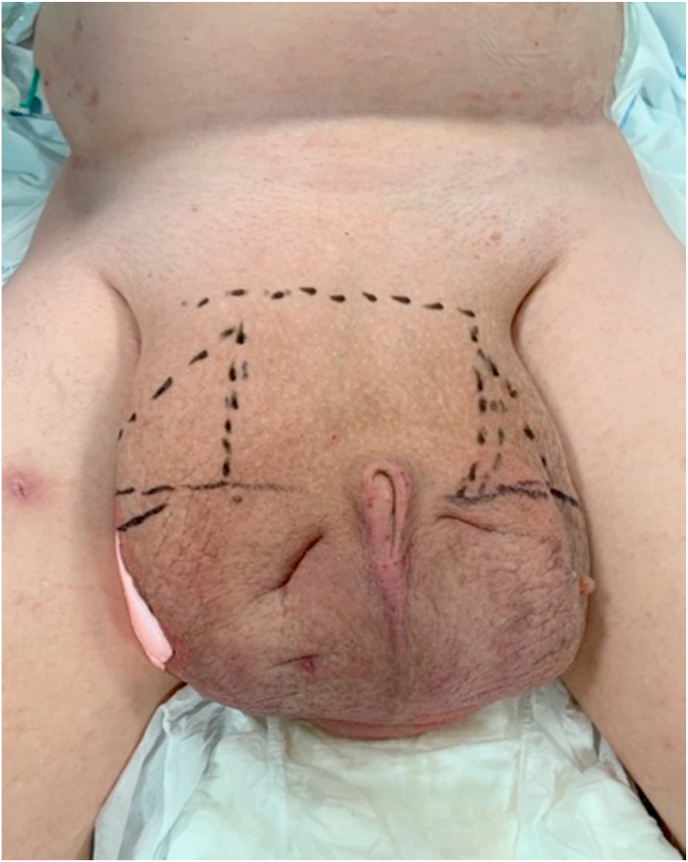
The scrotum before surgery. At this point, the patient could no longer walk due to discomfort from the increasing scrotum.

In March 2020 the patient was hospitalized with an inflammatory cutaneous scrotal infection which resolved under antibiotic treatment. A CT-scan showed that the majority of the bladder, a part of the sigmoid colon and a piece of the omentum was situated in the left inguinoscrotal hernia. (Fig. 1). Four months later the patient was hospitalized again with elevated infection parameters, this time with a subcutaneous scrotal abscess. The abscess was incised under local anesthesia and subsequently healing was obtained during of course of intravenous antibiotic treatment. The risk of a potentially uncontrollable infection in the area was considered high, and surgery on vital indication was planned (Fig. 2). The patient had decisional making capacity and was together with his mother thoroughly informed of the risks of intervention, before he agreed for treatment.

The cardiac condition was assessed for surgery. The patient was classified as NYHAIII with a blood oxygen saturation of 76% without oxygen and 88% with 4 L of nasal oxygen/min. The echocardiography showed a non-restrictive perimembranous VSD of 2,4 cm with a bidirectional shunt. The approximate anesthesia-related mortality risk was estimated to 20% by cardiology/anesthesiology specialists.

The surgery was planned as a joint venture surgery with a urologist and a plastic surgeon. The procedure started with a cross-incision under the penile opening. On the left side a hydrocele containing 500 ml of fluid was found, and after complete mobilization it was depleted, before the edges of the hydrocele-sack were sewn together behind the spermatic cord known as Winkelmann's technique. The hernia-sack with the bladder embedded in the wall was isolated, and carefully pushed back to the abdominal cavity. During the procedure it was decided to remove the left testicle due to the size of the inguinal hernial port, to reduce the risk of remission. Bassini-sutures closed the port, then a prolene-mesh was attached to the back of the external fascia and inguinal ligament and at last the external fascia was closed. On the right side the same method was used, except for the right testis was saved and the inguinal canal restored with a mesh repair of the posterior wall.

The skin of the penile shaft appeared surprisingly unaffected and could be preserved. An incision was made from the base of the penile shaft up in the abdominal midline to reposition the penis. Resection of excess skin to both sides were made with the approach to maintain the blood supply of the shaft skin. Diathermy and ligation was used for thorough hemostasis. Procedure time was 4 hours and 27 minutes, and blood loss was 400 ml.

**Fig. 3:**
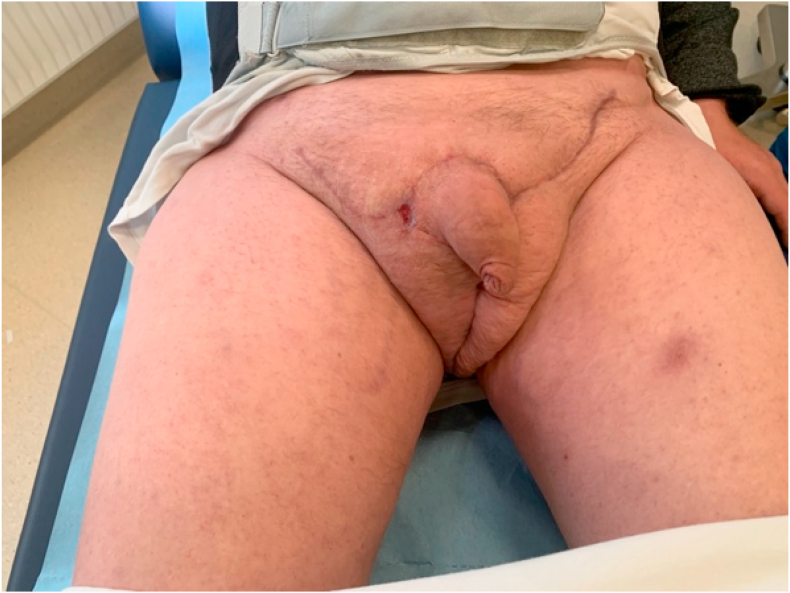
Check up and wound care was performed continuously in the plastic surgery outpatient clinic until pre-hernia habitual condition. The final result four months post-surgery.

After surgery the patient was at the ITU for three days before transferred to the department of plastic surgery. Final follow up was performed four months post-surgery; the patient was voiding naturally, and the scrotal appearance and penile function had been restored (Fig. 3).

## Discussion

There are no standard surgical procedures for the treatment of GISH and various techniques have been reported. A categorization has been made, depending on the size and the choice of surgery of the hernia. In relation to this, our patient had a type I hernia (Type I GISH when sac extends below mid inner thigh and mid between mid inner thigh and suprapatellar lines.). Our Case supports previous literature with hernioplasty being possible without resection of contents and being handled as one-step-surgery compared to type II (Type II GISH when sac extends below mid between mid inner thigh and suprapatellar lines and superior border of patellar bone.) and 10.13039/501100000032III (Type 10.13039/501100000032III GISH when sac extends below superior border of patellar bone).^2^ Considering our patient's comorbidity and perioperative mortality risk this was very important.

Surgical treatment of GISH containing the urinary bladder has to our knowledge only been described in the literature with successful outcome once before.^3^ As similar to our Case orchiectomy was also needed, why preoperative examination of the testis should always be performed.^2^

Important technical points in the surgical treatment described in the literature, is the complete dissection of all involved tissue-compartments and the use of scrotal advancement flaps from areas with normal, non-edematous skin.^4^ Our initial plan was to reconstruct the skin of the penile shaft with a skin graft as described in the literature as Charles’ Procedure.^5^ Fortunately, in our Case the penile skin appeared unaffected, and no skin graft was needed.

## Conclusion

We present a successful treatment of a GISH in a patient suffering from Trisomy 21 and Eisenmenger's syndrome We present a successful treatment of a GISH.

### Consent

Written informed consent was obtained from the patient for publication of this Case report including clinical pictures.

## References

[1] Prochotsky A., Dolak S., Minarovjech V. (2017). Giant inguinoscrotal hernia repair. Bratisl Lek Listy.

[2] Trakarnsagna A., Chinswangwatanakul V., Methasate A. (2014). Giant inguinal hernia: report of a Case and reviews of surgical techniques. Int. J. Surg. Case Rep..

[3] Tarchouli M., Ratbi M.-B., Bouzroud M. (Nov. 2015). Giant inguinoscrotal hernia containing intestinal segments and urinary bladder successfully repaired by simple hernioplasty technique: a Case report. J Med Case Rep.

[4] Lin T., Lin Y., Wu Y. (Apr. 2019). Penoscrotal edema: a Case report and literature review. BMC Urol.

[5] Hassan K., Chang D.W. (Dec. 2020). The Charles procedure as part of the modern armamentarium against lymphedema. Ann Plast Surg.

